# Contrast‐enhanced ultrasound repeatability for the measurement of skeletal muscle microvascular blood flow

**DOI:** 10.1113/EP091034

**Published:** 2023-02-04

**Authors:** Eleanor J. Jones, Philip J. Atherton, Mathew Piasecki, Bethan E. Phillips

**Affiliations:** ^1^ Centre of Metabolism, Ageing and Physiology, MRC‐Versus Arthritis Centre for Musculoskeletal Ageing Research and National Institute for Health Research (NIHR) Nottingham Biomedical Research Centre University of Nottingham Derby UK

**Keywords:** blood flow, contrast enhanced ultrasound, repeatability, skeletal muscle

## Abstract

Contrast‐enhanced ultrasound (CEUS) can be used to directly assess skeletal muscle perfusion. However, its repeatability over time has not yet been validated and therefore its use in longitudinal measures (i.e., exploring the impact of a chronic intervention or disease progression) is limited. This study aimed to determine the repeatability of CEUS for the measurement of skeletal muscle microvascular blood flow (MBF) at baseline and in response to exercise, across independent assessment sessions. Ten healthy volunteers (five female; 30 ± 6 years) had CEUS of the right vastus lateralis recorded in two separate sessions, 14 days apart. Measurements were taken at baseline, during an isometric leg extension and during recovery. Acoustic intensity data from a region of interest were plotted as a replenishment curve to obtain blood volume (*A*) and flow velocity (β) values from a one‐phase association non‐linear regression of mean tissue echogenicity. Linear regression and Bland–Altman analyses of *A* and β values were performed, with significance assumed as *P* < 0.05. Strong positive correlations were observed across sessions for all *A* and β values (both *P* < 0.0001). Bland–Altman analysis showed a bias (SD) of −0.013 ± 1.24 for *A* and −0.014 ± 0.31 for β. A bias of 0.201 ± 0.770 at baseline, 0.527 ± 1.29 during contraction and −0.203 ± 1.29 at recovery was observed for *A*, and −0.0328 ± 0.0853 (baseline), −0.0446 ± 0.206 (contraction) and 0.0382 ± 0.233 (recovery) for β. A strong agreement between CEUS MBF measures across independent sessions suggests it to be a repeatable method for assessing skeletal muscle perfusion over time, and therefore facilitates wider use in longitudinal studies.

## Abstract (highlights)

- **What is the central question of this study?**
  Contrast‐enhanced ultrasound (CEUS) can be used to directly assess skeletal muscle perfusion but its day‐to‐day repeatability over time has not yet been validated: is CEUS a repeatable method for the measurement of skeletal muscle microvascular blood flow (MBF) at rest and in response to exercise, across independent assessment sessions?
- **What is the main finding and its importance?**
  A strong agreement between CEUS MBF measures across sessions suggests it is a repeatable method for assessing skeletal muscle perfusion over time. This validation provides confidence for incorporating these measures into longitudinal studies such as a chronic intervention or disease progression to gain further knowledge of skeletal muscle microvascular function.

## 1 INTRODUCTION

Contrast‐enhanced ultrasound (CEUS) can assess real‐time microvascular blood volume (MBV) and blood flow velocity (MFV), facilitating determination of total microvascular blood flow (MBF) (Wei et al., 1998). CEUS uses ultrasound to measure the acoustic backscatter of intravascular gas‐filled microbubbles, which are stabilised by an outer lipid capsule. Tissue perfusion can be assessed through destruction of these microbubbles using high mechanical index (MI) ultrasound energy and the subsequent assessment of microbubble reappearance in a region of interest (ROI).

Unlike other methods of recording blood flow such as Doppler ultrasound and near infrared spectroscopy, CEUS can directly measure skeletal muscle perfusion as opposed to large vessel blood flow and oxygenation, respectively (Casey et al., 2008; Nioka et al., 2006). To date, CEUS has been used in humans to investigate skeletal muscle microvascular function in response to nutrition, exercise and disease (Amarteifio et al., 2011; Krix et al., 2010). Skeletal muscle is highly vascularised, which is functionally important for its contractile and metabolic actions particularly when metabolic demand increases during, for example, exercise. Additionally, poor perfusion and resultant ischaemia potentially underlie a number of metabolic situations observed in conditions such as type 2 diabetes as well as in ageing, including impaired glucose uptake and reduced muscle protein synthesis, both of which affect muscle function (Adelnia et al., 2019; Groen et al., 2014). As such, the ability to determine tissue perfusion in skeletal muscle enables further understanding of microvascular function in situations of health, disease and in response to environmental stimuli (i.e., exercise, nutrition and pharmaceutical agents).

However, one of the main limitations of CEUS for the assessment of skeletal muscle perfusion is the lack of validation for its application over time (Thomas et al., 2015). As such, most skeletal muscle CEUS assessments are performed as a single measure or used to assess responsiveness to an acute stimulus such as feeding (Abdulla et al., 2022) or acute exercise (Krix et al., 2010). CEUS does have potential to be used to study skeletal muscle perfusion in response to chronic intervention periods (e.g., longer‐term nutritional interventions), but confirmation of its repeatability over time, including in situations where the underlying tissue composition may alter (i.e., with hypertrophic or atrophic) would enable more widespread use. Therefore, the aim of this study was to determine the repeatability of CEUS across different sessions to assess microvascular blood flow in the vastus lateralis (VL) muscle.

## 2 METHODS

### 2.1 Ethical approval

Ten healthy young volunteers (five female; 30 ± 6 years) gave written informed consent to participate in this study, which was approved by the University of Nottingham Faculty of Medicine and Health Sciences Research Ethics Committee (FMHS 516‐2003). This study conformed with the latest version of the *Declaration of Helsinki*, except for database registration. Participant characteristics are summarised in Table 1. Before enrolling in the study all potential participants attended for a screening visit where medical history, height and weight, electrocardiogram (ECG) and blood pressure were recorded to confirm suitability for the study, with exclusion criteria including a body mass index (BMI) <18 or >35 kg/m^2^ or a history of cardiovascular, respiratory or neuromuscular disorders. All participants were non‐smokers not taking any vasoactive medications. All participants were asked to refrain from exercise for 48 h prior to each assessment visit and to maintain their physical activity and dietary intake profiles for the duration of the study. The menstrual cycle was not controlled for in female participants, but this has previously been shown not to affect microvascular function (Williams et al., 2020).

**TABLE 1:** Participant characteristics (*n* = 10).

Characteristic	Mean (SD)
Age (years)	30 (6)
Height (m)	1.73 (0.08)
Weight (kg)	70.7(14.8)
BMI (kg/m^2^)	23.4 (2.8)
VL CSA (cm^2^)	24.4 (9.7)
VL thickness (cm)	2.5 (0.58)

### 2.2 CEUS

Participants attended for their first study visit at ∼09.00 h after a 12 h overnight fast (water ad libitum). Images to determine VL cross sectional area (CSA) and thickness were recorded from the right leg at the start of this visit using ultrasound (MyLab, Esaote, Italy) and analysed using ImageJ software (NIH, Bethesda, MD, USA) as described previously (Inns et al., 2022).

**FIGURE 1:**
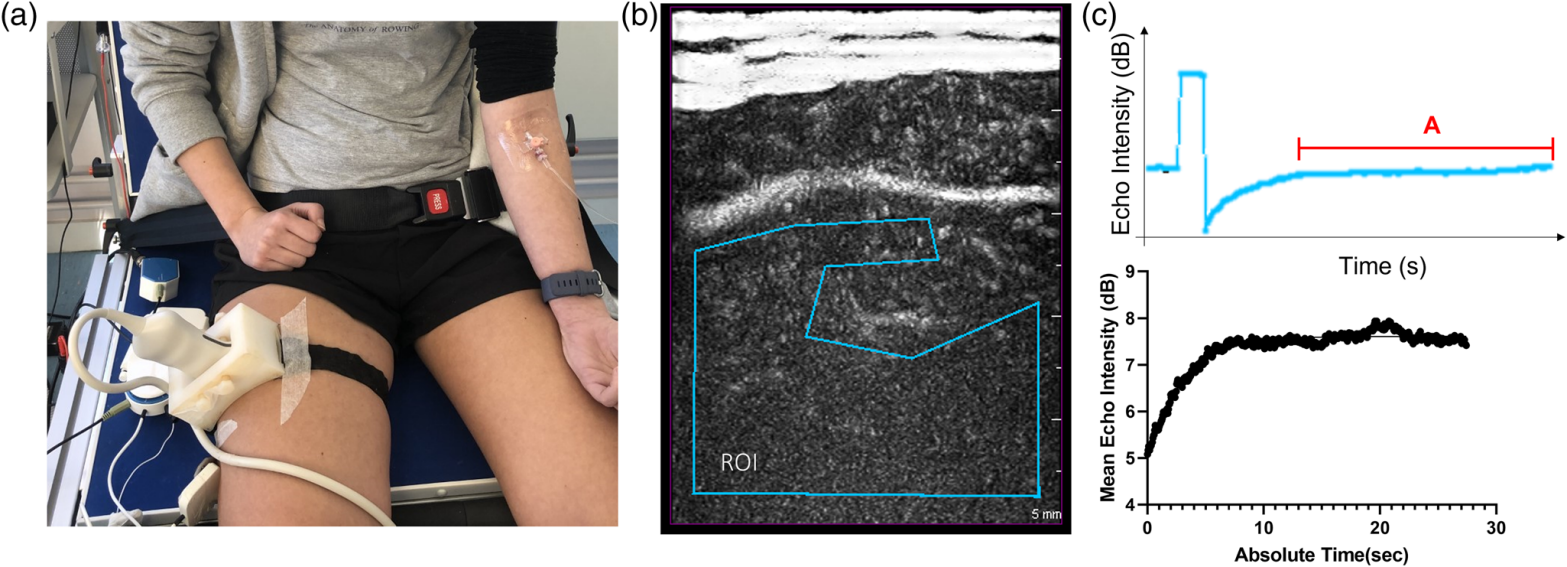
(a) Participant setup for the contrast enhanced ultrasound (CEUS) recordings. (b) A representative image showing a selected region of interest (ROI) avoiding rapid filling vessels. (c) Representative replenishment curve showing the *A* value (microvascular blood volume) from the plateau and one phase linear association to calculate *A* and β values.

MBF was measured in the right VL using a Philips iU22 ultrasound machine and L9‐3 probe (Philips Healthcare, Bothell, WA, USA) secured over the mid‐belly of the muscle by a custom housing unit (Figure 1a). The machine settings in ‘contrast‐mode’ were a low MI of 0.08 for replenishment capture and a high MI of 1.2 for the microbubble destruction ‘flash’. 2D gain was set at 95%, frame rate at 21 Hz and a contrast resolution of C30. A 20‐min rest period was completed before MBF assessments to allow pH and temperature equilibrium between the skin and the probes. Full details of this method have been published previously (Mitchell et al., 2013).

Two vials of SonoVue™ contrast agent (Bracco, Milan, Italy) were infused at 2 ml/min for 1 min and then 1 ml/min thereafter for a total of 10 min via a cannula situated in the medial cubital vein. Steady‐state concentration of microbubbles in the muscle vasculature was achieved within ∼2 min (Mitchell et al., 2013). Microvascular replenishment was recorded by three 30 s captures each beginning with a flash, to generate a replenishment curve. CEUS capture‐flash recordings were taken over a 1.5 min baseline period, during the first 30 s, middle 30 s and final 30 s of a 3 min submaximal isometric leg extension and during a 1.5 min recovery period immediately post‐exercise. The same protocol was then repeated 2 weeks later at the same time of day, with the probe in the same position facilitated by on‐skin location markings.

### 2.3 Data analysis

CEUS video recordings were exported to Q‐Lab software (Philips) to generate acoustic intensity data from an ROI selected to minimise signal contribution from connective tissue and large rapid‐filling vessels (Abdulla et al., 2022), with the same ROI parameters applied to each video for a participant (Figure 1b). Values were plotted as a replenishment curve to obtain *A* (blood volume) and β (flow velocity) values from a one phase association non‐linear regression of mean tissue echogenicity values (Figure 1c). MFV was calculated by measuring the reperfusion rate of the microbubbles in the muscle microvasculature following destruction with a high MI flash, given the infusion of contrast was continuous. Measurement of the concentration of microbubbles at steady state provided an estimation of microvascular cross‐sectional area or MBV. Linear regression and Bland–Altman analyses of the *A* and β values were performed (Prism (v9.2), GraphPad Software, Inc., San Diego, CA, USA) on combined values and separate data from baseline, the first 30 s of the contraction, and recovery time points. Significance was assumed when *P* < 0.05.

## 3 RESULTS

### 3.1 Participant characteristics

The mean age of the participants was 30 ± 6 years (Table 1) with other anthropometric characteristics presented in Table 1. There were equal numbers of male and female participants (5).

### 3.2 CEUS repeatability

**FIGURE 2:**
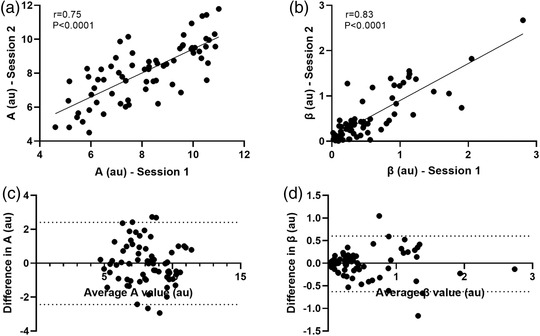
Linear regression plots of pooled data for *A* (microvascular blood volume) (a) and β (microvascular blood flow velocity) values (b) and Bland–Altman plots of *A* (microvascular blood volume) (c) and β (microvascular blood flow velocity) values (d) from two repeat contrast enhanced ultrasound (CEUS) sessions of the vastus lateralis muscle. Bias and 95% limits of agreement represented by dashed lines.

A strong positive correlation of pooled data was observed between the two sessions for both the *A* (*r* = 0.75; *P* < 0.0001; Figure 2a) and β (*r* = 0.83; *P* < 0.0001; Figure 2b) values. Bland–Altman analysis showed a bias (±SD) of −0.013 ± 1.24 (95% limits of agreement: −2.4, 2.4) for *A* (Figure 2c) and −0.014 ± 0.31 (−0.63, 0.60) for β (Figure 2d).

**FIGURE 3:**
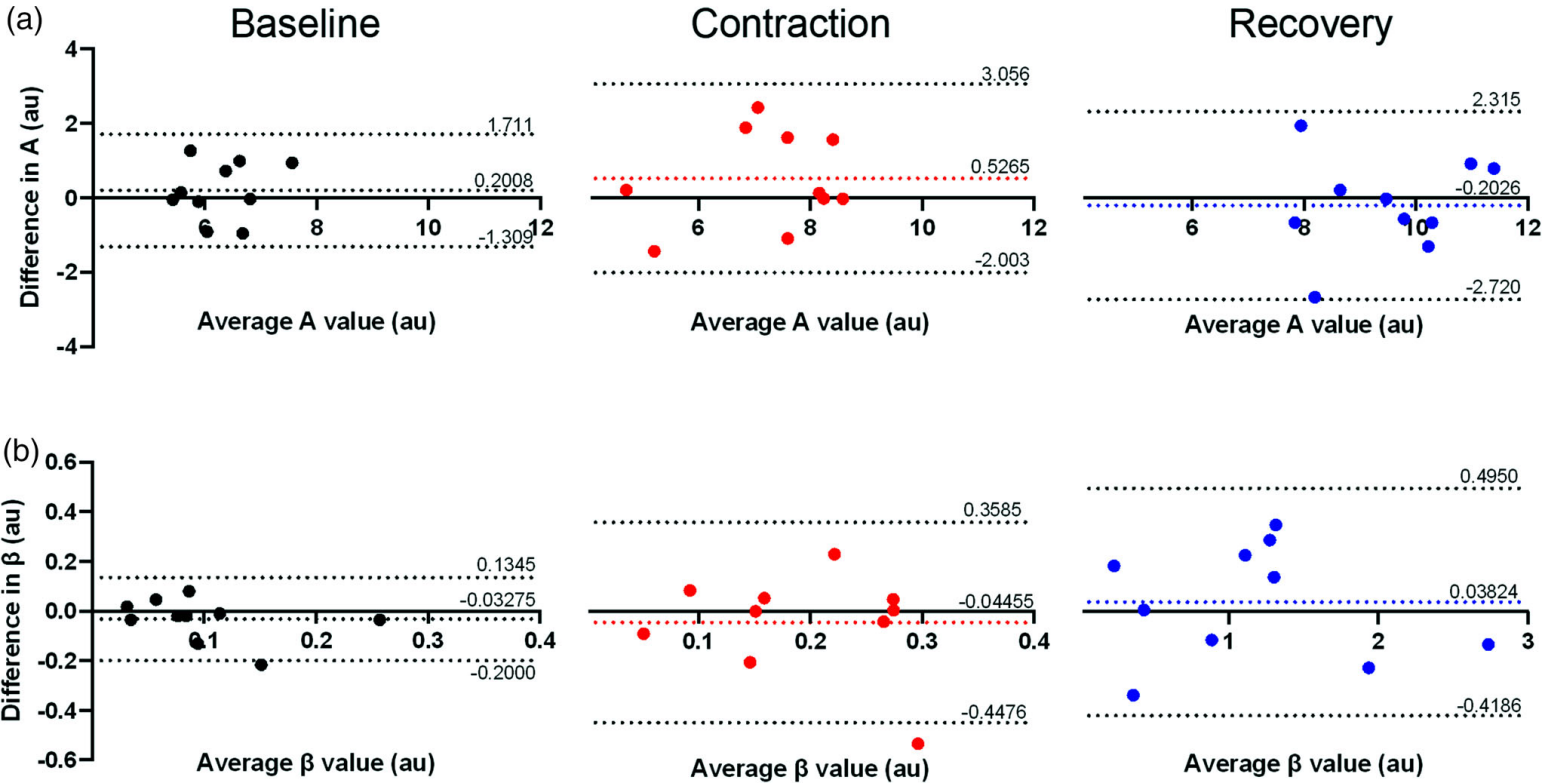
Bland–Altman plots of individual data for *A* (microvascular blood volume) (a) and β (microvascular blood flow velocity) (b) values at baseline (black), during contraction (red) and at recovery (blue) from two repeat contrast enhanced ultrasound (CEUS) sessions of the vastus lateralis muscle. Bias and 95% limits of agreement represented by dashed lines.

When separated for individuals and for the different conditions, Bland–Altman analysis showed a bias of 0.201 ± 0.770 (−1.31, 1.71) at baseline, 0.527 ± 1.29 (−2.00, 3.06) during contraction and −0.203 ± 1.29 (−2.72, 2.32) at recovery for *A* (Figure 3a). For β, a bias of −0.0328 ± 0.0853 (−0.2, 0.135) was observed at baseline, −0.0446 ± 0.206 (−0.448, 0.359) during contraction and 0.0382 ± 0.233 (−0.429, 0.495) at recovery (Figure 3b).

## 4 DISCUSSION

This study demonstrates a strong agreement between CEUS‐derived measures of vastus lateralis MBF across two independent sessions, 14 days apart. This agreement was maintained at rest, during a voluntary contraction and during recovery.

The *A* value obtained from the plateau phase of the replenishment curve is representative of MBV and showed significant correlation and agreement between the sessions. This indicates repeatability of the measures across the two sessions, in addition to during the different physiological conditions of rest, muscle contraction and recovery. The *A* value is commonly used when reporting skeletal muscle perfusion, as probe position has less influence than for the β value (Mitchell et al., 2013).

The β value represents MFV, and although the values showed greater variability, there was strong correlation between the two sessions reported herein. This further supports the repeatability of CEUS to measure muscle blood flow parameters over time. However, for CEUS measures to be repeatable the probe must be positioned in the same place to ensure the same capture area and enable the transfer of the analysis ROI across captured images from separate sessions. Although greater MFVs were reported during the recovery phase this was consistent across sessions, as were values during contraction and at rest, demonstrating CEUS is a reliable method to identify MBF changes during muscle activity over time.

Skeletal muscle blood flow is an important physiological parameter to study due to its role in muscle function per se and in mal/adaptation with, for example, disease and changes in activity status. The agreement of MBF measures across the different scenarios of rest, contraction and recovery demonstrate that this method can be used to study both acute and longitudinal changes in MBF as a result of muscle activity, such as during and after fatigue, and subsequently compared to function with the ability of simultaneous recording. CEUS is currently under‐utilised in the research environment but offers the benefit of directly measuring muscle blood flow using a technique that is easy to use, relatively cost effective and reliable. However, this method is not suitable for use during dynamic movements because accurate measurement is reliant on maintaining an ROI which is free of artefact (i.e., connective tissue and rapid‐filling vessels) across images (Mitchell et al., 2013). Similarly in situations of gross alterations in muscle architecture, ROI selection may have to be adapted to ensure that subsequent ROIs of the same size do not transgress muscle boundaries or necessitate the inclusion of non‐muscle tissue (e.g., bone).

To conclude, CEUS is a repeatable method for longitudinally measuring MBF in static skeletal muscle in situations where there is no profound change in muscle architecture (i.e., extreme atrophy). This validation provides confidence for incorporating these measures into longitudinal studies to gain further knowledge of skeletal muscle microvascular function with, for example, a chronic nutritional intervention or disease progression.

## AUTHOR CONTRIBUTIONS

Eleanor J. Jones, Philip J. Atherton, Mathew Piasecki and Bethan E. Phillips contributed to the conception and design of the work. Eleanor J. Jones acquired the data. Eleanor J. Jones analysed the data. Eleanor J. Jones and Bethan E. Phillips drafted the manuscript and prepared the figures. Eleanor J. Jones, Bethan E. Phillips and Mathew Piasecki contributed to the interpretation of the results. All authors contributed to the revision of the manuscript. All authors have read and approved the final version of this manuscript and agree to be accountable for all aspects of the work in ensuring that questions related to the accuracy or integrity of any part of the work are appropriately investigated and resolved. All persons designated as authors qualify for authorship, and all those who qualify for authorship are listed.

## CONFLICT OF INTEREST

All authors declare no conflicts of interest.

## Supporting information

Statistical Summary Document

## Data Availability

The data that support the findings of this study are available from the corresponding author upon reasonable request.
